# Navigating Europe's agricultural transition: Systemic policy approaches to mixed farming and agroforestry

**DOI:** 10.12688/openreseurope.20398.1

**Published:** 2025-05-27

**Authors:** Holger Pabst, Johannes Lang, Simone Sterly

**Affiliations:** 1 Institute for Rural Development Research, Frankfurt am Main, Germany

**Keywords:** Mixed farming, agroforestry, Common Agricultural Policy (CAP), efficiency, resilience, sustainability, agri-food system

## Abstract

Mixed farming and agroforestry systems offer the potential to optimise resource use and reduce environmental impacts by integrating crops, livestock and trees. By improving soil health, biodiversity, and carbon sequestration, these diversified farming systems can help to build sustainable, resilient and climate-smart adapted agri-food systems and make farms more resilient to climate change. However, barriers to widespread adoption include financial constraints, knowledge gaps, and regulatory barriers.

To support the transition to more sustainable agri-food systems, policymakers need to align CAP support with sustainability goals. Simplifying regulations and strengthening research, knowledge-sharing networks, and farmer training will enable the implementation and optimal management of these systems. Cooperation between farms can improve circularity and resource efficiency, including at landscape level. The public goods provided by sustainable agriculture need to be remunerated to ensure viability. Increasing consumer awareness and integrating mixed farming products into mainstream value chains can provide remuneration in the markets, but public funding for sustainable farming practices is likely to remain necessary.

As CAP reforms continue, the integration of mixed farming systems into agricultural landscapes will be crucial for long-term environmental and economic resilience. The success of these reforms will determine how well European agriculture adapts to climate challenges while ensuring food security and ecosystem sustainability.

## Introduction

European agriculture is at a crossroads, facing increasing pressures from climate change, biodiversity loss, economic uncertainties, and changing societal expectations. The ongoing transformation of the European Union’s (EU) Common Agricultural Policy (CAP) aims to address these challenges by promoting greater sustainability, resilience, and competitiveness in the farming sector. A milestone in this transition was the European Commission's (EC) Strategic Dialogue on the Future of EU Agriculture ^1^. Bringing together key stakeholders, it has fostered a broad consensus on the need for a CAP that better supports farmers, enhances sustainability, and strengthens rural communities. The report further emphasizes that without adaptation, farming in some regions could become unsustainable due to climate-induced challenges. Hence, adaptation measures will need to be strengthened at both farm and landscape scales to improve resource efficiency, ecosystem resilience and economic stability ^1^. In this context, the Strategic Dialogue calls for systematic support to assist farmers in implementing resilient farming systems and highlights the role of diversified farming models ^1^, such as Mixed Farming and Agroforestry Systems (MiFAS). However, at least partly at odds with the Strategic Dialogue is Commissioner Hansen's Vision for Agriculture and Food ^2^ presented in early 2025. This roadmap focuses on the attractiveness, competitiveness and innovation of the agricultural sector and is less oriented towards the sustainability aspects mentioned in the final report of the Strategic Dialogue.

The authors define MiFAS as diverse farm activities that integrate crop, forestry, and livestock production within a field, farm, or landscape, to optimize resource use through synergies, collaboration and diversification. This occurs, for example, when by-products or outputs from one activity serve as inputs for another, such as using manure to fertilize crops. The farm-level includes diversified farming systems like agroforestry and integrated farming, while landscape-level mixedness involves cooperation between farms, improving circularity beyond individual farms ^3^. Overall, this increases sustainability by improving resource use and land use efficiency compared to more specialized systems ^4, 5^. MiFAS therefore have the potential to increase farm resilience and promote circular resource use, contributing to the EU's agricultural sustainability goals.

Nevertheless, farmers and the wider agri-food system are facing substantial challenges in the transition to and continued management of MiFAS. The transition requires cultural shifts as well as systemic changes that need financial investments and infrastructure development ^6^. Once critical thresholds of the transition are reached, some barriers, such as value chain adjustments and availability of specialised machinery, may lessen over time. The continuous management of established MiFAS faces trade-offs between economic and environmental objectives. Balancing profitability for farmers with long-term sustainability remains difficult and requires fair compensation for public goods such as biodiversity and climate resilience. Key transition challenges identified in workshops across 10 countries were technical issues, knowledge gaps, profitability concerns, supply chain constraints, regulatory barriers, lack of public funding, and cultural and societal challenges ^6^. While these may not be specific to MiFAS, there are broad gaps and challenges that need to be addressed to create a policy environment conducive to MiFAS.

But what might such a conducive policy environment look like? In order to answer this question, the authors analysed the challenges outlined above and formulated potential recommendations. Through workshops with relevant stakeholders and stakeholder networks, potential barriers, gaps and opportunities were identified, while further dialogue and interactive online sessions led to the identification of policy objectives, key supporting factors and existing policy instruments. A final policy workshop in Brussels with stakeholders from policy, administration, civil society, and farming organisations aligned the recommendations with current political debates and decision-making processes.

**Figure 1.**
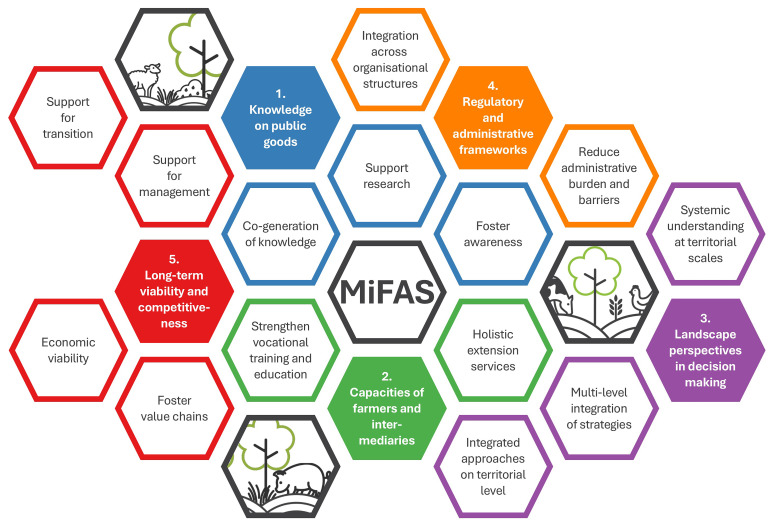
Schematic illustration of the five identified policy areas for a transition to sustainable, resilient and efficient MiFAS.

The work was undertaken as part of the EU-funded MIXED project ^7^ and this contribution presents the project's perspective accordingly. The present article provides policy recommendations that outline ways to better anchor MiFAS in the political dialogue, and in this way align EU agricultural policies with the urgent need for sustainable and resilient food systems. Through the integration of these approaches, the CAP and its implementation in Member States can evolve into a more adaptive and forward-looking framework, ensuring that European agriculture can thrive in the face of future challenges. The presented policy recommendations share a common vision for a sustainable, diversified and resilient agricultural system with the White Paper Transforming European Food Systems with Agroforestry ^8^. In line with the Strategic Dialogue on the Future of EU Agriculture ^1^, the recommendations are mutually supportive and are calling for actions at EU and Member States level to develop supportive policies for the uptake of MiFAS across Europe. Five policy areas need to be tackled to promote the transition to MiFAS ( Figure 1).

## Knowledge on public goods

The Strategic Dialogue identifies knowledge gaps on sustainability metrics and the provision of public goods in different farming models and emphasises the need for benchmarking systems ^1^. This is also evident for MiFAS, as their performance in terms of efficiency, resilience and sustainability is highly context-specific ^3– 5^, and knowledge of the long-term benefits of different MiFAS is lacking. This lack of knowledge perpetuates a system that encourages specialisation and prevents appreciation of the benefits of mixed farming. To effectively value and remunerate the public goods provided by MiFAS, stakeholders must first understand MiFAS and acknowledge how they benefit society – both are still lacking among the public as well as among policymakers and administrators. Still, evidence-based policymaking requires a transdisciplinary perspective and consolidated knowledge of the conditions under which MiFAS deliver public goods.

To address the knowledge gaps surrounding MiFAS, it is essential to strengthen research support through increased funding and extended project durations. This enhances understanding of MiFAS impacts, provision of public goods, and optimal management strategies. Transdisciplinary and landscape-scale research needs to be encouraged. Farmers need to be involved as equal partners and appropriately remunerated for on-farm, participatory and long-term trials. In order not to hamper research progress, regulatory exemptions for research should be considered.

Collaborative systems involving all stakeholders should be set up to improve knowledge sharing and uptake of MiFAS research. Resources should be allocated to on-farm trials, lighthouse farms and landscape-scale living labs to raise awareness and encourage mutual learning, e.g. through cross visits and field days. A structured knowledge-sharing network should not only link farmers, processors, retailers, consumers, scientists, policymakers and administrators, but also include an accessible database to strengthen synergies and practical application.

Communication campaigns can raise awareness of the public goods provided by MiFAS. Campaigns can address local issues, e.g. soil erosion and/or nitrate leaching, but should emphasize the benefits of mixed farming; for example, in terms of biodiversity, soil health, carbon sequestration, and the link to established values such as tradition and animal welfare. Campaigns should be transparent about the trade-offs involved, presenting both the benefits and drawbacks of mainstream and mixed farming systems.

## Capacities of farmers and intermediaries

As noted above, the Strategic Dialogue highlights the knowledge gap in diversified farming practices and states that current vocational training systems favour specialisation over holistic systems thinking ^1^. It calls for a review of education and training systems to support sustainable models of agriculture.

The prevalent specialisation paradigm in agriculture today results in deep but segmented knowledge among farmers and intermediaries, including vocational training institutions and extension services. In contrast to specialised agriculture, MiFAS depend on knowledge of a wide range of different farming practices. While knowledge of crop, livestock and fruit production is widely available but remains isolated or in silos, capacity building is increasingly important within the context of new, innovative but also traditional MiFAS that require specific as well as integrated knowledge ^6^.

In view of this, curricula in schools, colleges and universities should be revised to include broader, systems-based perspectives for farmers and related sectors. Courses should be offered to help farmers explore diversification and mixed farming options, and to equip them and their workers with the skills for implementation. In addition, the promotion and integration of traditional agricultural knowledge with systems thinking should be encouraged.

There is a need for holistic extension services. Farm advisors with both broad and specific knowledge, as well as administrative skills can develop plans tailored to each farm using systems thinking and considering the landscape level. The long-term planning periods of the agricultural sector need to be matched by funding for such holistic advisory services.

## Landscape perspective in strategies and decision making

Landscape level mixedness allows for the efficiency of specialised farming while improving circularity within a region, leading to greater sustainability. Interactions between farms can counteract specialisation tendencies of whole regions with negative consequences such as water eutrophication. The Strategic Dialogue recognises the importance of regional cooperation between farmers to counteract the negative effects of specialised agricultural landscapes ^1^. It emphasises that landscape level strategies can improve sustainability and economic viability. Such strategies to achieve mixedness on a landscape level are called for to be able to promote cooperation between farms and across value chains, and to overcome path dependencies. A landscape perspective also helps to identify potential synergies and constraints, to set objectives and to address problems of collective action ^4, 9^. Consequences that would be external to farm business considerations are, to varying degrees, internal in a landscape perspective. Thus, a shift to landscapes provides both the incentives and the means to address negative externalities of agriculture. In this context, it is important to bear in mind that trust, social cohesion and strong cooperation between all regional actors are the basis for achieving landscape-level objectives.

Long-term landscape strategies need to be jointly developed with all relevant stakeholders. They must not only account for specific regional needs and services along the value chain and in relation to agricultural practices affecting landscapes, but also consider overarching European policies and objectives, such as the Biodiversity Strategy, the Green Deal and the Farm-to-Fork Strategy – although the latter two are no longer mentioned in the current Vision for Agriculture and Food of the EU ^2^. 

Improving access to farming system data through public data repositories will help enable landscape perspectives and promote evidence-based setting of targets. This entails a need to bolster the capacity of regional actors to analyse landscape-level information, as well as supporting better stakeholder involvement and strengthening the interface between research and policy.

Territorial mechanisms with "landscape coordinators" should be established to facilitate interaction between farmers, as well as with other stakeholders. Equipped with tools to build trust, resolve conflicts and create cross-sectoral agreements, such coordinators will encourage collaboration to achieve synergies and regional landscape objectives.

## Regulatory and administrative frameworks

Administrative systems are often inadequate for MiFAS, as the diversity of farming practices within MiFAS leads to extensive bureaucracy and increased administrative burden ^6^. This is directly related to regulatory coherence, as a lack of integration across regulatory domains can create barriers. However, stakeholders can develop practical, economically viable and mutually beneficial solutions through collaboration and continuous dialogue. In addition, as agriculture, including MiFAS, is a long-term endeavour, policies need to ensure long-term stability and adaptability, as returns on investments can take decades. The Strategic Dialogue recognises regulatory fragmentation and administrative burden as significant barriers to sustainable agriculture and emphasises the need for a simplified regulatory framework ^1^. In the context of the transition to more mixed land use systems in Europe, it is important to note that such simplification must not be traded off against accountability and compliance with regulations that ensure overarching objectives such as biodiversity protection, nature conservation or climate change mitigation and adaptation.

Administration must improve their understanding of farm management and set up farmer-friendly frameworks to reduce the administrative burden for MiFAS operators. In addition, regulatory frameworks need to be sufficiently stable to allow for long-term commitments and investments, while being flexible enough to accommodate different MiFAS and ensuring compliance with environmental legislation. Also, responsive and concise communication between administrations, farmers and intermediaries must focus predominantly on key obligations.

Strengthened interdisciplinary organisations, such as levy boards or producer organisations may facilitate knowledge transfer and collaboration. These organisations can act as intermediaries and provide practical feedback to administrators and policy makers to improve the coherence of policies and the support for MiFAS. In addition, fast-track communication and decision-making structures should be established at national or sub-national levels across different regulatory domains. 

## Long-term viability and competitiveness

MiFAS may face significant challenges in achieving economic viability due to reduced economies of scale, increased labour requirements etc. This is also underlined by the Strategic Dialogue, which identifies economic viability as a key challenge for small and mixed farms and emphasises the need for targeted financial support ^1^. Profitability is often achieved long after the introduction of MiFAS, as some mixed systems require significant investment due to extensive changes in land management, purchase of new machinery and adaptation of production methods.

As of now, the range of public goods that MiFAS provide is not sufficiently remunerated by the markets. While there is some potential for specialised marketing to a sustainability-aware clientele, this approach has quantitative limitations. If the benefits of MiFAS are to be obtained at a larger scale, their respective products will have to be incorporated into existing processing and marketing channels that deal with large amounts of agricultural goods with less regard to their origin and method of production. Therefore, the realisation of the transformation of the European agricultural system must consider "the long-term investment cycles in the sector" and should be financed by both public and private capital ^1^.

In order to ensure the economic viability of practices that provide public goods, the existing support framework must be assessed and adapted to the objectives of the agri-food system transformation (e.g. public money for public goods principle). Investment support schemes that encourage mixed farming at both farm and landscape level are needed to meet the different needs for the establishment and management of MiFAS. Additional support to offset increased costs and reduced income during the transition period needs to be accompanied by criteria for agri-environmental schemes to compensate for economic disadvantages and to support collective action at the landscape level. Finally, the promotion of value chains for MiFAS products, such as through cooperatives, can strengthen market positions by pooling resources and knowledge, helping to offset the challenges faced by smaller production systems.

## Conclusion

Policy frameworks in European countries are diverse, influenced by unique historical, cultural and socio-economic factors. They often consider specificities at the sub-national level, in natural conditions, traditional farming practices and the resulting cultural landscapes. Due to this diversity, the policy recommendations provided here are rather general and aim to address common challenges and opportunities across Europe. They include considerations that must be part of, but do not encompass the whole extent of tailored, subsidiary policies that are called for.

Several stakeholder groups each have a crucial role to play in facilitating transitions to mixed practices at different levels, including farm, inter-farm and landscape levels. Ideally, intermediaries can take all perspectives into account and effectively communicate requirements between all stakeholders. Farmers need financial, technical, and knowledge support for the adoption of mixed systems. Financial support, training in diverse farming systems, and specialized advisory services will help. Digital tools can aid decision-making, while farmer-led research and peer exchanges will promote practical learning and adaptation. Advisors and extension services are vital for the adoption of mixed systems, requiring specialized training in areas like crop-livestock integration and agroforestry. On-farm training, demonstration projects, and knowledge-sharing platforms will help improve advisory support and provide best practices. Farmer cooperatives and networks can support by enabling resource-sharing, cost reduction, and joint investments in infrastructure. They enhance efficiency, sustainability, and access to funding, while fostering trust and developing sustainable value chains. Guided by sustainability indicators, policymakers must reform regulations, simplify procedures, and provide financial support for adoption. A CAP targeting mixed farming with long-term funding as well as regional policies should prioritize areas with the greatest potential to benefit from diversification. Research needs to prove the sustainability and economic viability of mixed farming and involve farmers in research trials, while revised agricultural curricula need to embrace systems thinking. Endorsing sustainably sourced products in public procurement, including those from mixed farming systems, will stabilise markets and encourage the transition. Retailers can support mixed farming by increasing demand for sustainably produced food through awareness, certification schemes, and direct-to-consumer models. Encouraging retailers to include such products and running public campaigns will further boost market demand. Landscape planners should integrate mixed agricultural systems into land-use strategies, focusing on regions facing environmental challenges, while incentives for landscape-level transitions and regional coordination can align mixed farming policies with local priorities and encourage farm cooperation.

Although there is still a need for more research on mixed farming systems, the article demonstrates that an effective and targeted support policy at national and sub-national level will be necessary to realise a transition in European agriculture.

## Outlook

This paper highlights the challenges of the transition to resilient MiFAS under the current policy framework. Key challenges appear to persist as the EU's 2023 CAP implementation unfolds. While preparations for the post-2027 CAP are already underway, the EU has published their Vision for Agriculture and Food ^2^. A roadmap that has been met with scepticism and criticism, particularly regarding the preservation and protection of natural resources and public goods ^10^. In the face of global developments, crises and geopolitical challenges, it remains to be seen to what extent the future of European agriculture will favour MiFAS. Considering the ongoing reform processes, the policy recommendations presented are timely and well positioned to inform the next CAP framework to support a more resilient, sustainable and integrated agricultural future for Europe.

## Data Availability

No data associated with this article.
