# Supplementary material for: Navigating Europe's agricultural transition: Systemic policy approaches to mixed farming and agroforestry
Source: Open Res Eur. 2026 Feb 10;5:142. Originally published 2025 May 27. [Version 2] doi: 10.12688/openreseurope.20398.2 (PMC13014087; doi:10.12688/openreseurope.20398.2)
Supplement: Peer review history

# Reviewer report: Reviewer response for version 2

**DOI:** 10.21956/openreseurope.24882.r69537

**Published:** 2026-03-24

**Authors:** Nicholas Jordan

**Affiliations:** 1 University of Minnesota, Saint Paul, Minnesota, USA

**Summary statement**: This open letter provides an integrated set of actions to support the expansion of Mixed Farming and Agroforestry Systems (MiFAS) as part of a program of sustainability transition for European agriculture. After outlining the merits of MiFAS agriculture, the letter presents a policy proposal encompassing five major focal points (knowledge on public goods, capacities of farmers and intermediaries, landscape perspectives in decision making, regulatory and administrative frameworks and long-term viability and competitiveness). The letter appears to be motivated by a belief that it is “…increasingly important to advocate for sustainability and resilience goals as part of the next CAP.”, because of various political processes that are tending to reduce emphasis on such goals.  

**Overview comment**:  In my opinion, this is an incisive policy proposal that provides a decently comprehensive account of leverage points to address systemic barriers to development and scaling of diversified farming systems that meet contemporary needs for multifunctional, resilient, and regenerative agroecosystems. I think that this concise review and synthesis of a broad policy agenda will be helpful to advocates and coalitions working on regional sustainability challenges.  The text struck me as lucid and polished. As a person working to advance agricultural diversification on regional scales, I found it useful: interesting, informative, and energizing!

 I do perceive a gap in the policy recommendations: policy related to organizing and supporting the dynamic processes of extensive scaling of Mixed Farming and Agroforestry Systems. Such scaling qualifies as a sustainability transition, I think. Supportive policy for the underlying processes of transition—according to current understanding of how sustainability transitions can be undertaken—would address matters such as support for transformative transdisciplinary initiatives in support of agricultural diversification, support for organization of regional transformation initiatives, support for public-sector leadership in organization and support for public/private/NGO cooperation on the timescales needed for sustainability transitions, and other measures in similar veins.

 The point is that such policy is key to create enabling conditions for range innovation and investments needed to pursue the policy agenda proposed by the authors. To add a broad account of this latter sort of policy would substantially enhance the scope of the article, and I don’t think that is necessary.  I do think it would be quite valuable for the authors to acknowledge that additional policy (and also policy experimentation and learning) will be necessary to support comprehensive implementation of the policy agenda that they have identified. Also, given the current situation – need for a comprehensive and systemic approach to supporting development and support, can the authors suggest any elements of strategy for organizing and implementing such an approach?

 I have several more minor comments.  First, I think it would be helpful for the authors to say a bit more about what their intentions are for this article.  What is the intended audience, and how might this audience be benefited by the contents of the article?  Second, and relatedly, it would helpful to the authors to be a bit more explicit about what knowledge gap(s) they are filling through this work.  Lastly, it would help readers, I think, if the authors were to provide a few specific examples of “modern versions of MiFAS, providing a viable approach to achieving the EU’s agricultural sustainability goals, including boosting rural employment and sustaining family farming.”

Where applicable, are recommendations and next steps explained clearly for others to follow? (Please consider whether others in the research community would be able to implement guidelines or recommendations and/or constructively engage in the debate)

Partly

Does the article adequately reference differing views and opinions?

Yes

Are all factual statements correct, and are statements and arguments made adequately supported by citations?

Yes

Is the rationale for the Open Letter provided in sufficient detail? (Please consider whether existing challenges in the field are outlined clearly and whether the purpose of the letter is explained)

Partly

Is the Open Letter written in accessible language? (Please consider whether all subject-specific terms, concepts and abbreviations are explained)

Yes

Reviewer Expertise:

agroecology; agricultural diversification; sustainability transitions; innovation and scaling systems.

I confirm that I have read this submission and believe that I have an appropriate level of expertise to confirm that it is of an acceptable scientific standard, however I have significant reservations, as outlined above.

# Reviewer report: Reviewer response for version 2

**DOI:** 10.21956/openreseurope.24882.r69395

**Published:** 2026-02-25

**Authors:** Rosemary Venn

**Affiliations:** 1 Coventry University, Coventry, England, UK

I appreciate the edits made by the authors and am happy for this to now be indexing.

Where applicable, are recommendations and next steps explained clearly for others to follow? (Please consider whether others in the research community would be able to implement guidelines or recommendations and/or constructively engage in the debate)

Yes

Does the article adequately reference differing views and opinions?

Partly

Are all factual statements correct, and are statements and arguments made adequately supported by citations?

Yes

Is the rationale for the Open Letter provided in sufficient detail? (Please consider whether existing challenges in the field are outlined clearly and whether the purpose of the letter is explained)

Partly

Is the Open Letter written in accessible language? (Please consider whether all subject-specific terms, concepts and abbreviations are explained)

Yes

Reviewer Expertise:

NA

I confirm that I have read this submission and believe that I have an appropriate level of expertise to confirm that it is of an acceptable scientific standard.

# Reviewer report: Reviewer response for version 1

**DOI:** 10.21956/openreseurope.22075.r59966

**Published:** 2025-09-27

**Authors:** Rosemary Venn

**Affiliations:** 1 Coventry University, Coventry, England, UK

This open letter champions Mixed Farming and Agroforestry Systems (MiFAS) as a critical approach to Europe’s agricultural transition, arguing the many benefits these mixed systems have on social, economic and environmental resilience. The letter positions itself at the intersection of diverging policy agendas seen in the European Commission’s Strategic Dialogue on the Future of EU Agriculture, and Commissioner Hansen’s Vision for Agriculture and Food. The letter draws on findings from the Horizon 2020 MIXED project as well as ongoing policy debate and negotiations. The authors identify five key areas for policymakers to focus on, which they argue will improve farmer uptake of MiFAS: knowledge on public goods, capacities of farmers and intermediaries, landscape perspectives in decision making, regulatory and administrative frameworks and long-term viability and competitiveness. These recommendations are situated within other Horizon 2020 project results such as AGROMIX and are supportive of each other.

 The letter goes on to unpack the five key policy areas needed to promote the transition to MiFAS, with actions and recommendations put forward for different stakeholders (farmers, communities, policy makers), taking into consideration various barriers to transition. There is some discussion on the cost and / or feasibility of the recommendations put forward, but more could be included. The open letter is timely and relevant to ongoing discussions around CAP reform (post 2027), as well as local action needed.

 If the authors unpacked a little more of the diverging policy interests and contexts, the case for MiFAS could be more strongly made and indeed, the rationale for the open letter made clearer. Not all readers will be aware of how significant the subtle shift away from the Strategic Dialogue and Green Deal for Europe is. Additionally, spending a bit more time in the text considering issues around productivity of MiFAS in the long term (vis a vis conventional agriculture) with climate projects would make the case for the MiFAS stronger.

 ​​​​​​Suggestions:

- Make the policy context clearer in the introduction, so the rationale of the open letter is more obvious.
- Setting the scene for non-MiFAS practitioners would be helpful and bring the open letter into scope of more readers. There is assumed knowledge on how these systems work, why they are no longer the norm, as well as their current support or lack of in agricultural policies. It might be helpful to include some land use figures for MiFAS as well as where in Europe these systems are best suited for development and support. There could be a link to 2025’s summer heatwaves and fires discussed, positioning MiFAS in this context as climate resilient and adaptation.
- Including links to other key policies could give this open letter further reach and impact. I note this is also a suggestion from the first reviewer who mentions Nature Restoration Law. I agree with this and also suggest including or providing clear links to the EU Soil Strategy (and forthcoming Soil Monitoring Law) and European Climate Law.

Where applicable, are recommendations and next steps explained clearly for others to follow? (Please consider whether others in the research community would be able to implement guidelines or recommendations and/or constructively engage in the debate)

Yes

Does the article adequately reference differing views and opinions?

Partly

Are all factual statements correct, and are statements and arguments made adequately supported by citations?

Yes

Is the rationale for the Open Letter provided in sufficient detail? (Please consider whether existing challenges in the field are outlined clearly and whether the purpose of the letter is explained)

Partly

Is the Open Letter written in accessible language? (Please consider whether all subject-specific terms, concepts and abbreviations are explained)

Yes

Reviewer Expertise:

Transitions to sustainable food systems via agroecology, agroforestry land use policy

I confirm that I have read this submission and believe that I have an appropriate level of expertise to confirm that it is of an acceptable scientific standard, however I have significant reservations, as outlined above.

# Response

**Published:** 2026-01-27

**Authors:** Holger Pabst

Responses to the Reviewers comments: **General remarks**: Reviewer: *More unpacking of diverging policy interests for clearer rationale and context of the open letter*

- Authors: The majority of the work on this open letter was carried out in spring 2025, based on EU documents relevant at the time, including those on the strategic dialogue and Commissioner Hansen's vision. Since then, discussions on the multiannual financial framework and the CAP 2027 have progressed inexorably, including some far-reaching and drastic changes (e.g. consolidation in the new fund, abandonment of the pillar principle and budget reductions) that could not have been foreseen at the time, at least not to this extent. Due to these fundamental changes in the political discussion, and given that this is a constantly changing and volatile field, the authors do not consider it advisable to revise the open letter or the methodology the closed MIXED project. Instead, we view the open letter as reflecting the status quo as of spring 2025. However, the introduction now includes a short paragraph regarding the aforementioned changes and discussions at EU level.

Reviewer: *More consideration of MiFAS productivity*

- Authors: We agree with the reviewer that MiFAS's productivity will be a decisive factor in determining the extent to which, and the speed at which, a transition can or will take place. We have therefore added some explanation to the paragraph on long-term viability and competitiveness. Unfortunately, the MIXED project does not provide any concrete information on the long-term productivity of the examined MiFAS, partly because the issue of productivity was not addressed in such detail. Nevertheless, we believe that the additional notes emphasise the importance of considering long-term productivity. We’ve also included an explanation of the diverging efficiencies in terms of labour and of non-labour resources in the introduction, which are major factors for productivity.

**Reviewer's suggestions**: Reviewer: *Clearer policy context in the introduction for a more obvious rationale of the open letter*

- Authors: See answer to general remarks and the changes on EU-Level

Reviewer: *More explanations around MiFAS*

- Authors: In the introduction, we added some short explanation of the traditionality of MiFAS, with some examples, and that they are more and more falling behind today’s technical agriculture – but also economically wise. Now bringing together new technique and tools with this old knowledge might lead to new MiFAS with multiple benefits to the environment but also economies. MiFAS encompass a multitude of different land use systems, although it would be nice to have, it is not possible to put this into single numbers, maps, etc. The authors are also of the opinion of it not being feasible to speak of areas best suited for MiFAS development. As an assumption, the most could be in already diverse agricultural landscapes, whereas the diversity reflects also the agricultural infrastructure as well as the processing and markets sectors.

Reviewer: *Better refer to other key environmental policies.*

- Authors: The authors consider this point raised by both reviewers to be valid. However, given the numerous changes in the environmental and agricultural fields at the EU level since spring 2025, it is debatable whether referencing legal acts such as the NRL or the EU Soil Strategy would enrich the article. Given the current volatile political environment and declining focus on environmental and climate targets within the EU and worldwide, the authors have decided against incorporating such references in detail. However, the revised version briefly mentions possible synergies with relevant legislation in the fields of nature conservation and the environment. See chapter on landscape perspective in strategies.

# Reviewer report: Reviewer response for version 1

**DOI:** 10.21956/openreseurope.22075.r54853

**Published:** 2025-07-26

**Authors:** Elsa Varela

**Affiliations:** 1 Spanish National Research Council, Institute of Public Goods and Policies, Madrid, Community of Madrid, Spain

This open letter discusses the role of Mixed Farming and Agroforestry Systems (MiFAS) in Europe’s transition toward more sustainable, resilient agricultural landscapes. The authors argue that MiFAS offer significant environmental and social benefits, including improved resource efficiency, biodiversity conservation, and climate adaptation. The paper identifies five key policy areas necessary to support the transition: knowledge on public goods, capacities of farmers and intermediaries, landscape-level strategies, regulatory and administrative frameworks, and long-term economic viability. The recommendations draw on findings from the Horizon 2020 MIXED project and are positioned as timely contributions to ongoing debates around the EU Common Agricultural Policy (CAP) and the European Green Deal.

 Overall, this is a well-structured and timely manuscript that provides relevant insights for policymakers, researchers, and practitioners working on agri-environmental policy and mixed farming. The paper is accessible and synthesizes a large body of work, including project findings and stakeholder dialogues.

**Use of Citations and Examples**

 While most statements and arguments are supported by references (notably to the MIXED project deliverables and EU policy documents), some sections—particularly those relating to behavioural barriers, payment schemes, and value chain development—would benefit from additional examples and citations to strengthen credibility. 

**Overall Assessment**

 This is a relevant and well-organized paper that addresses a timely topic and contributes useful policy recommendations. I provide some detailed comments below aimed to make the article more robust and practically valuable.

 I look forward to seeing a revised version that incorporates these suggestions.

**Detailed comments and suggestions**

**1. Contextualization of the Extent and Potential of MiFAS**

 The introduction would benefit from a clearer quantification of the current prevalence and distribution of MiFAS in Europe, as well as estimates of their potential expansion.This context would allow readers to understand both the baseline and the opportunity for increasing MiFAs land uses.

**2.** **Framing of innovation and competitiveness**

 The manuscript at times appears to frame sustainability and innovation/competitiveness as competing priorities. In practice, innovation—including technical, organizational, and social innovation—will be essential for MiFAS to remain economically viable and it can be a driver of both sustainability and competitiveness. Consider adding examples of innovative practices or technologies that enhance MiFAS performance (e.g., precision grazing, collaborative machinery use).

**3.** **Labour Intensity and Family Farming**

 The letter notes that MiFAS are more labour-intensive but does not link this to broader structural trends, such as the decline of family farms. I would maybe consider briefly discussing whether support for MiFAS could also help sustain family farming structures and rural employment.

 4. **Target audience for communication campaigns**

 The manuscript recommends communication campaigns but does not specify their target audience. Please clarify whether these campaigns are aimed at the general public, policymakers, supply chain actors, or multiple audiences. This will help readers assess the feasibility and intended impact of such campaigns.

**5. Opportunities under the Nature Restoration Law**

 The authors mention a number of relevant policies, but I woudl like to draw their attention to the recently passed NRL. I reckon that to enhance the policy relevance of their letter, the NRL can be at least briefly mentioned, since it introduces binding targets and funding mechanisms that may be relevant for MiFAS to create additional incentives or funding streams for MiFAS transitions.

**6. Behavioural factors affecting shift towards sustainable practices**

 The manuscript emphasizes structural and economic factors but less so behavioural aspects that can play a key role in farmers' adoption of sustainable practices. I would briefly reflect on how behavioural and cultural factors shape adoption, potentially citing relevant literature (e.g., Barnes et al. 2019 on behavioural change in agri-environmental schemes).

**7. Indicators and result-based payments**

 The authors note the importance of developing indicators for policy making. This links to transforming also the payment schemes and monetary incentives towards more result-based payments. Referring briefly to this shift towards result and not only practice -based payments would also address the need for stronger evidence and examples supporting statements about incentives.

**8. Value Chains and Market Integration**

 There appears to be some inconsistency between integrating MiFAS products into existing value chains and developing dedicated value chains. Please clarify how these approaches might be sequenced or combined. Are dedicated value chains envisioned as transitional measures before mainstream integration?

**9. Definition of Intermediaries**

 The term “intermediaries” is used in the conclusions without specifying which actors this includes. Please, clarify which organizations or individuals are considered intermediaries (e.g., cooperatives, advisory services, supply chain coordinators).

Where applicable, are recommendations and next steps explained clearly for others to follow? (Please consider whether others in the research community would be able to implement guidelines or recommendations and/or constructively engage in the debate)

Yes

Does the article adequately reference differing views and opinions?

Yes

Are all factual statements correct, and are statements and arguments made adequately supported by citations?

Partly

Is the rationale for the Open Letter provided in sufficient detail? (Please consider whether existing challenges in the field are outlined clearly and whether the purpose of the letter is explained)

Yes

Is the Open Letter written in accessible language? (Please consider whether all subject-specific terms, concepts and abbreviations are explained)

Yes

Reviewer Expertise:

Agroforestry systems; Economic valuation of public goods; socioeconomic dimensions of wildfires; farmer's adoption of agro-environmental schemes

I confirm that I have read this submission and believe that I have an appropriate level of expertise to confirm that it is of an acceptable scientific standard, however I have significant reservations, as outlined above.

## References

[1] : The role of behavioural factors and opportunity costs in farmers' participation in voluntary agri‐environmental schemes: A systematic review. *Journal of Agricultural Economics* .2023;74(3) : 10.1111/1477-9552.12538 617-660 10.1111/1477-9552.12538

# Response

**Published:** 2025-08-15

**Authors:** Holger Pabst

Dear Elsa, Thank you for providing this valuable review of our article — you certainly have some valid points. Please note that we are currently waiting for the second review before we start revising the open letter. Regards, Holger

# Response

**Published:** 2026-01-27

**Authors:** Holger Pabst

Responses to the Reviewers comments: **General remarks:** Reviewer: *More use of external citations for better credibility.*

- Authors: We added more scientific citations where feasible.

**Reviewer’s suggestions:** Reviewer: *Contextualization of the extent and potential of MiFAS*

- Authors: In the introduction, we added some short explanation of the traditionality of MiFAS, with some examples, and that they are more and more falling behind today’s technical agriculture – but also economically wise. Now bringing together new technique and tools with this old knowledge might lead to new MiFAS with multiple benefits to the environment but also economies. MiFAS encompass a multitude of different land use systems, although it would be nice to have, it is not possible to put this into single numbers, maps, etc. The authors are also of the opinion of it not being feasible to speak of areas best suited for MiFAS development. As an assumption, the most could be in already diverse agricultural landscapes, whereas the diversity reflects also the agricultural infrastructure as well as the processing and markets sectors

Reviewer: *Framing of innovation and competitiveness*

- Authors: The authors did not intend to present sustainability and innovation/competitiveness as competing priorities. Rather, they see them as mutually beneficial. We have therefore rephrased the relevant paragraphs where appropriate.

Reviewer: *Labour Intensity and Family Farming*

- Authors: We have included this in a section of the introduction that also clarifies what we mean by traditional MiFAS and where we try to clarify the relation of traditional and modern ones.

Reviewer: *Target audience for communication campaigns*

- Authors: We revised the respective paragraph to make it clearer that the target audience and the intended recipients of the information campaigns may vary depending on the context and content of the campaign.

Reviewer: *Opportunities under the Nature Restoration Law*

- Authors: The authors consider the point raised by both reviewers to be valid. However, given the numerous changes in the environmental and agricultural fields at the EU level since spring 2025, it is debatable whether referencing legal acts such as the NRL or the EU Soil Strategy would enrich the article. Given the current volatile political environment and declining focus on environmental and climate targets within the EU and worldwide, the authors have decided against incorporating such references in detail. However, the revised version briefly mentions possible synergies with relevant legislation in the fields of nature conservation and the environment. See paragraph regarding landscape perspective in strategies and reference to the NRL as well as other environmental policies.

Reviewer: *Behavioural factors affecting shift towards sustainable practices*

- Authors: We have now noted that in addition to cultural factors, behavioural ones are also relevant to adoption, as conceptualised in the project as a knowledge-intention-action process, and reference as the source a deliverable from the MiXED project.

Reviewer: *Indicators and result-based payments*

- Authors: Although we would welcome the idea of a shift towards more results-oriented funding, we deliberately did not elaborate on this approach in the article. This is mainly because there are currently few results-oriented funding measures in place. This, in turn, is linked to the problem of verification, which is based on sufficient knowledge of the relevant agricultural practices. We therefore focused on the recommendation to expand research, as we consider this to be a prerequisite for any results-oriented funding. Nevertheless, the establishment of such funding remains questionable in view of the numerous and complex MiFAS. The conclusion referred to sustainability indicators. To avoid misunderstandings and achieve greater clarity, this was changed to objectives.

Reviewer: *Value Chains and Market Integration*

- Authors: We hope to have clarified this by spelling out that both approaches are necessary and complementary; we cannot, however, speak to specifics in sequencing or combination, as we have no data on that and suspect they will be highly individual to value chains, What is clear is that marketing MiFAS products as specialty, niche products, while it has advantages in terms of appreciation and prices that can be realised, is not scalable to what is needed in the face of the necessary transition of the agri-food system.

Reviewer: *Definition of Intermediaries*

- Authors: This is a valid point. The reviewer is right in both points, a) the term intermediaries was not clear enough, and b) the term relates advisory and extension services and cooperatives, etc. We revised the text accordingly.
